# Prenatal PM_2.5_ Exposure and Its Association with Low Birth Weight: A Systematic Review and Meta-Analysis

**DOI:** 10.3390/toxics12070446

**Published:** 2024-06-21

**Authors:** Nichapa Parasin, Teerachai Amnuaylojaroen, Surasak Saokaew

**Affiliations:** 1 School of Allied Health Science, University of Phayao, Phayao 56000, Thailand; nichapa.pa@up.ac.th; 2 School of Energy and Environment, University of Phayao, Phayao 56000, Thailand; 3 Atmospheric Pollution and Climate Change Research Units, School of Energy and Environment, University of Phayao, Phayao 56000, Thailand; 4 Division of Social and Administrative Pharmacy, Department of Pharmaceutical Care, School of Pharmaceutical Sciences, University of Phayao, Phayao 56000, Thailand; surasak.sa@up.ac.th; 5 Unit of Excellence on Clinical Outcomes Research and Integration (UNICORN), School of Pharmaceutical Sciences, University of Phayao, Phayao 56000, Thailand; 6 Center of Health Outcomes Research and Therapeutic Safety (Cohorts), School of Pharmaceutical Sciences, University of Phayao, Phayao 56000, Thailand

**Keywords:** prenatal exposure, PM_2.5_, particulate matter, air pollution, low birth weight, birth outcomes, pregnancy, preterm birth

## Abstract

Exposure to PM_2.5_ while pregnant is associated with negative effects on low birth weight (LBW). This study employed a systematic review and meta-analysis to investigate the impact of PM_2.5_ exposure during pregnancy on LBW. A search of databases such as Scopus, ScienceDirect, and PubMed identified thirteen appropriate studies. This study used a random-effects model to calculate pooled odds ratios (ORs) and 95% confidence intervals (CIs) for each trimester. The findings revealed a significant relationship between PM_2.5_ exposure and LBW in both the first and second trimesters (OR 1.05, 95% CI 1.00–1.09, *p* < 0.001). There was no significant difference between trimesters (*p* = 0.704). The results emphasize the persistent influence of PM_2.5_ on fetal development throughout all stages of pregnancy. Reducing air pollution is critical for improving pregnancy outcomes and decreasing the incidence of LBW. Further study is needed to improve exposure assessments and investigate the underlying biological pathways.

## 1. Introduction

Fine particulate matter (PM_2.5_) is a significant global health issue. PM_2.5_ is a complicated mixture of particles that have various chemical compositions. These particles come from several sources, such as burning, car emissions, industrial activities, and natural occurrences in the environment [1,2]. It can deeply infiltrate the respiratory system and enter the bloodstream, leading to many negative health consequences [1,3]. Pregnant women and children may be more vulnerable to these effects [4], such as low birth weight (LBW) [5], increased risk of childhood obesity [6], autism spectrum disorder (ASD) [7], intrauterine growth restriction (IUGR) [8], giving birth before the due date [9], and poor neurodevelopment [10,11]. Exposure to PM_2.5_ can exacerbate these issues in children, potentially causing long-term health and developmental problems [1]. Infants with LBW are more likely to experience fatalities as newborns, have developmental problems, and suffer from long-term health problems.

While knowledge of the mechanisms is limited, a growing body of studies has emphasized the importance of both environmental and genetic factors in the development of LBW [12,13]. The potential mechanisms proposed include oxidative stress, inflammation, placental malfunction, endocrine disruption, and epigenetic alterations [14,15,16,17]. According to a previous study, air pollution exposure in pregnant women might interfere with the mitochondrial function of the fetus, disrupt metabolic pathways, and negatively affect the fetus’s growth and development [18].

There is growing evidence indicating a relationship between air pollution and adverse pregnancy outcomes, specifically LBW [18,19,20,21]. For example, Toro-Heredia et al. [14] found a correlation between exposure to PM_2.5_ during pregnancy and an increased risk of delivering a child with LBW. Volk et al. [19] assessed the potential influence of PM_2.5_ on the growth and development of the fetus [20]. Their study revealed a direct correlation between being exposed to PM_2.5_ and an increased risk of developing LBW. Kalkbrenner et al. [18] found a strong correlation between being exposed to PM_2.5_ during pregnancy and an increased risk of having children with LBW. Raz et al. [20] found a significant correlation between exposure to PM_2.5_ while pregnant and negative birth outcomes, emphasizing the critical significance of the prenatal period. In addition, Liu et al. [21] conducted a thorough evaluation and statistical analysis, which supports the relationship between prenatal exposure to particulate matter (PM) and LBW at full term in developed countries. Furthermore, Andersen et al. [22] investigated the effects of prolonged exposure to PM and found a substantial negative correlation with LBW. Ghosh et al. [23] found a link between exposure to ambient and household PM_2.5_ and lower birth weight and gestational age, both of which are associated with higher rates of neonatal and infant mortality, especially in low- and middle-income countries. However, it is critical to recognize the complexities in understanding the overall impact of air pollution on birth outcomes. The interaction between genetic predispositions and environmental exposures is crucial, since both inherited susceptibilities and environmental circumstances contribute to the development of LBW [24,25]. The complexities of this situation underscore the necessity for additional study to improve our understanding of how environmental stressors, specifically air pollution, affect fetal development and the outcomes of childbirth.

The objective of this study is to address the lack of evidence by conducting a systematic review and meta-analysis to investigate the effect of prenatal exposure to PM_2.5_ on the risk of LBW. In contrast to previous studies, this work distinctively emphasizes noteworthy effects throughout all trimesters, elucidating the specific influences of PM_2.5_ on fetal development throughout each stage of pregnancy. Furthermore, this study aims to improve the reliability and precision of our findings by incorporating data exclusively from cohort and cross-sectional studies. This study presents compelling data on the relationship between PM_2.5_ exposure and LBW, and it aims to provide a thorough understanding of the key periods of exposure and their impact on fetal development by analyzing data from multiple trimesters. The result of this study provides crucial information for public health initiatives aimed at shaping policies to reduce air pollution and protect vulnerable populations, particularly pregnant women and their prenatal infants.

## 2. Materials and Methods

### 2.1. PECO

The primary objective of this study is to investigate the impact of PM_2.5_ exposure during pregnancy on LBW. We used the PECO framework to design the following research question: “What are the effects of exposure to PM_2.5_ during pregnancy on the weight at birth and the health outcomes from the time the baby is in the womb until early childhood?” This study focuses on the first, second, and third trimesters of pregnancy. The group under consideration includes pregnant women of all ages, from adolescents to elderly mothers, from several socioeconomic backgrounds, and with a variety of health concerns. This study also focuses on PM_2.5_. The comparison group in this study consists of people with similar demographic and health characteristics who have been exposed to lower levels of PM_2.5_ or have not been exposed to any. The current evaluation is low birth weight (LBW), which is defined as a weight below 2500 g at birth. This research followed the standards outlined in the PRISMA statement for systematic reviews, which may be found at www.prisma-statement.org (accessed on 17 June 2024), and it was registered with the International Prospective Register of Systematic Reviews (PROSPERO) under registration number CRD42024549059.

### 2.2. Search Strategy

We conducted a search on three databases including PubMed, Scopus, and ScienceDirect. The search covered publications from January 2000 to December 2023 to ensure an examination of the most recent and relevant findings. In addition to database searches, we manually searched the reference lists of relevant articles to find any additional studies. To ensure a thorough search, we also examined the gray literature, which includes conference abstracts, theses, and reports from health organizations. The investigation was carried out using a blend of keywords and Boolean operators. The search covered several keywords pertaining to air pollution, with a specific emphasis on particulate matter (PM_2.5_) and its influence on birth weight and health outcomes. To expand the range of the search, phrases were merged using the Boolean operator “OR.” The search phrases used were “PM_2.5_”, “particulate matter”, “air pollution”, “prenatal exposure”, “pregnancy exposure”, “birth weight”, “low birth weight”, and “preterm birth”. The phrases were merged using the logical operator “OR” to ensure a thorough search for pertinent studies. To focus the search on the specific demographic and outcomes, the terms “prenatal”, “pregnancy”, “fetus”, “newborn”, “infant”, and “early childhood” were employed. In addition, the notions of “health outcomes” and “fetal development” were combined using the Boolean operator “OR” to include related ideas. To cover studies investigating the relation between PM_2.5_ exposure and the stated outcomes, the terms “association”, “risk”, “impact”, and “effect” were incorporated as keywords. To ensure that the results were accurate and applicable, the searches were restricted to academic articles written in English and published.

### 2.3. Study Selection

This study included both cohort and cross-sectional studies that focus on prenatal PM_2.5_ exposure and its effects on newborns, with participants of all genders and ethnicities. The main objective was to evaluate the impact of pregnancy-related exposure to PM_2.5_ on LBW during the perinatal period. The primary variable of concern was birth weight, specifically LBW, defined as being less than 2500 g at the time of birth. Both full-term and preterm births were included in this analysis. Studies that explicitly distinguished between full-term LBW and preterm LBW were particularly noted to ensure accurate and detailed analysis. To ensure that the data were relevant, this study focused on English language articles. Eligible studies had to use air pollution monitoring data, land-use regression models, or satellite-based estimates to evaluate the average exposure to PM2.5. Only studies that used statistical metrics such as odds ratios (ORs), relative risks (RRs), or hazard ratios (HRs) alongside their accompanying confidence intervals were considered. Exclusion criteria applied to studies that focused on populations other than pregnant women and their children, such as adults or non-human subjects. We excluded studies that did not evaluate PM_2.5_ exposure or focused solely on indoor air pollution unless they were directly related to outdoor air quality. Letters, comments, case reports, editorials, unpublished theses, conference abstracts, review papers, opinion pieces, and non-peer-reviewed articles were not eligible for consideration. Finally, this review only included studies with complete papers.

### 2.4. Outcome Measurement

This study calculated ORs and 95% CIs to evaluate the correlation between exposure to PM_2.5_ and the potential of LBW. The measurement of LBW varied across the studies included in the analysis. Both full-term and preterm LBW were considered, and studies that explicitly reported on full-term LBW were noted. This distinction was crucial for ensuring that the analysis accurately reflected the impact of PM_2.5_ exposure on different categories of LBW. Additionally, we assessed whether the original studies accounted for confounding factors such as maternal age, socioeconomic status, smoking status, pre-existing health conditions, and other environmental exposures. We extracted and considered this information in our analysis to provide a comprehensive understanding of the impact of PM_2.5_ exposure. Some studies used self-reported data from mothers, while others relied on medical records, birth registries, and hospital databases to obtain information on birth weight. Several data sources contribute to the reliability and comprehensiveness of the findings on LBW. These measures provide a consistent evaluation of the magnitude and direction of the association. The present study included articles evaluating LBW using widely accepted diagnostic criteria. LBW is defined as a birth weight that is less than 2500 g. A preterm birth occurs before 37 weeks of gestation. We conducted an assessment to compare and analyze the effects of different levels of PM_2.5_ exposure during pregnancy on vital health indicators in various populations. This allowed us to better understand the effects of prenatal PM_2.5_ exposure on these critical health indicators. We used the I^2^ statistic to assess the degree of heterogeneity observed across the study findings. This statistical metric measures the degree to which differences in characteristics, rather than chance occurrences, explain the total variability observed in the studies. Furthermore, we performed Egger’s regression test, a statistical method that assesses the relationship between effect estimates and their related variances, to investigate the potential presence of publication bias. By employing a rigorous methodology, we effectively measured and gained a detailed understanding of the relationship between PM_2.5_ exposure during pregnancy and negative birth outcomes. In STATA/SE software version 14.2 (StataCorp LLC, College Station, TX, USA), a meta-analysis technique was used to merge and examine the available data. The analysis of binary outcomes involved calculating pooled odds ratios, or relative risks, along with 95% CIs. This study evaluated the variation across the publications using the I^2^ statistic and then examined any possible bias in publishing through a funnel plot analysis and Egger’s test. In the presence of significant heterogeneity, this study employed a random-effects model to extrapolate the results beyond the included studies, acknowledging that factors such as variations in population demographics, methods of exposure assessment, and research designs may cause the reported effects to vary. By utilizing this model, this meta-analysis offers more resilient and dependable estimates of the correlation between prenatal PM_2.5_ exposure and LBW. We achieved this by considering a wider variety of study settings, thereby improving the relevance of the findings to various situations.

### 2.5. Data Extraction and Quality Assessment

To ensure accuracy and consistency, a systematic data extraction method was used. Two authors independently collected data from each study using a standardized form that included information about the study design, demographic characteristics, exposure assessment, and measured outcomes. When disagreements arose between the two authors, they resolved them through deliberation and, if necessary, sought advice from a third author to reach a consensus.

The Newcastle–Ottawa Scale (NOS) [26] was used to assess the quality of the studies included in the analysis. The NOS is divided into three primary domains as follows: selection, comparability, and result assessment. The scoring of each study was determined according to the following criteria:

1. Selection (0–4 stars): Evaluation of exposure, selection of the non-exposed cohort, representativeness of the exposed cohort, and proof that the outcome of interest was absent at the beginning of the trial.
2. Comparability (0–2 stars): Evaluation of the confounding variables, such as age, socioeconomic status, and health issues.
3. Outcome (0–3 stars): Evaluation of the result, length of the monitoring period, and sufficiency of the monitoring of the groups.

The maximum score for each study was 9 stars, with higher values signifying higher methodological quality. The utilization of the NOS facilitated a uniform and unbiased evaluation of the quality of the studies, offering a strong foundation for this meta-analysis.

### 2.6. Ethical Considerations

As this study is a systematic review and meta-analysis based on public data, it did not require the participation of human beings. However, our study was approved by the University of Phayao Human Ethics Committee (HREC-UP-HSST 1.1/034/67). The systematic review and meta-analysis processes were conducted following the standard methodologies specified in the PRISMA statement.

## 3. Results

### 3.1. Study Selection and Characteristics

**Figure 1:**
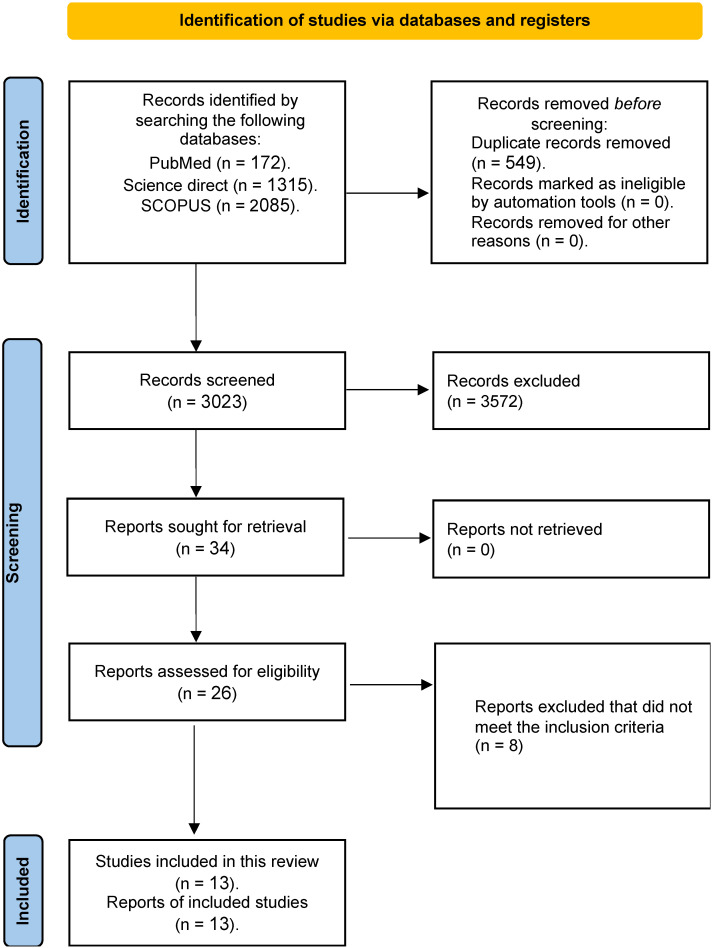
Prisma flow diagram of this study.

We used exclusion criteria to reduce the selection of studies from an initial pool of 3572 to focus on the most related studies. After removing 3023 duplicates, the selection decreased to 549 articles for further consideration. Using the inclusion criteria—studies investigating the effect of prenatal PM_2.5_ exposure on LBW—523 articles were excluded from consideration. As a result, a total of 26 full-text articles were evaluated for eligibility because of their accessibility, relevance, and full-text availability. Studies that exclusively examined populations other than pregnant mothers and their children were excluded from our analysis. Furthermore, studies that failed to conduct direct measurements or evaluations of PM_2.5_ were also excluded. To give precedence to original research findings, opinion articles, editorials, review papers, conference abstracts, and unpublished theses were excluded from the compilation. Non-peer-reviewed articles and various forms of non-original research, including case reports, letters, and comments, were excluded from the analysis. The final selection included 13 peer-reviewed studies that reported original research findings on the impact of prenatal PM_2.5_ exposure on LBW. These data are visually represented in the PRISMA flow diagram illustrated in Figure 1. The publication dates of the 13 studies incorporated in the analysis span from 2013 to 2023, as indicated in Table 1. Sample sizes for the studies incorporated in the analysis varied between 1410 and 3,389,450 individuals, for an aggregate dataset comprising 6,643,272 persons. In addition to Asia and Europe, the investigations were carried out in the United States and other regions.

### 3.2. PM_2.5_ Exposure and LBW Diagnostic

This study evaluates and analyzes existing studies to investigate the relationship between exposure to PM_2.5_ during pregnancy and the occurrence of LBW. The analysis involves examining data gathered from a wide range of cohort and cross-sectional studies conducted in different geographic regions, such as Asia, Europe, and the United States. This study largely focuses on pregnant mothers and their children, with sample sizes ranging from 1410 to 3,389,450 people. This study primarily focuses on pregnant women and children, utilizing methods to evaluate LBW based on widely accepted diagnostic criteria. LBW is defined as a birth weight of less than 2500 g based on the World Health Organization (WHO) guidelines [40]. For example, preterm birth is defined as birth occurring before 37 weeks of gestation, while full-term birth occurs between 37 and 42 weeks of gestation [41]. We conducted an evaluation to determine the impact of PM_2.5_ during critical stages of growth and development, including several trimesters of gestation. The evaluation of PM_2.5_ exposure was assessed using several techniques such as geographic information systems (GISs), air-quality monitoring data, land-use regression models, and satellite-based exposure algorithms.

### 3.3. Quality Assessment

We used the NOS, a well-established measure used in meta-analysis to evaluate the methodological quality of non-randomized trials (as shown in Table 2). This enabled an evaluation of the overall quality of the papers that were included in the analysis. The NOS criteria categorize the possible nine points into three categories as follows: outcome (three points), comparability (two points), and selection (four points). Several studies received a score of four points, indicating the highest possible rating for the purpose of selection. This finding indicates that the methods used for cohort selection and reducing selection bias were reliable and robust. In a similar vein, a considerable number of the studies received the highest possible score of two points in the comparability category. This indicates that they effectively controlled for crucial variables that might have distorted the association between PM_2.5_ exposure and LBW. Nevertheless, some variability was identified within the category of outcomes. With a few exceptions, most investigations obtained a rating of two out of a possible three points in this particular category. The principal factor contributing to the diminished ratings is uncertainty regarding the extent and adequacy of follow-up, a critical component when assessing the enduring consequences of PM_2.5_ exposure on LBW. For example, the outcome category scores of Rodríguez-Fernández et al. [27] and Balidemaj et al. [37] were diminished as a result of inadequate follow-up periods. The range of total NOS scores documented in the research was six to nine. The highest possible score of nine was attained by studies including those by Zhang et al. [28], Zhou et al. [32], Zhang et al. [33], Shang et al. [36], and Zhou et al. [35], which signifies a considerable degree of methodological excellence. Additional studies, including those by Balidemaj et al. [37] and Rodrguez-Fernández et al. [27], obtained lower scores because of certain constraints in their evaluations of outcomes. The acknowledged uncertainties in the outcome category are denoted by asterisks (*) beneath these scores, which significantly reduces the reliability of the data provided by these studies.

### 3.4. Result and Discussion

#### 3.4.1. Impact of Prenatal PM_2.5_ Exposure on LBW

**Figure 2:**
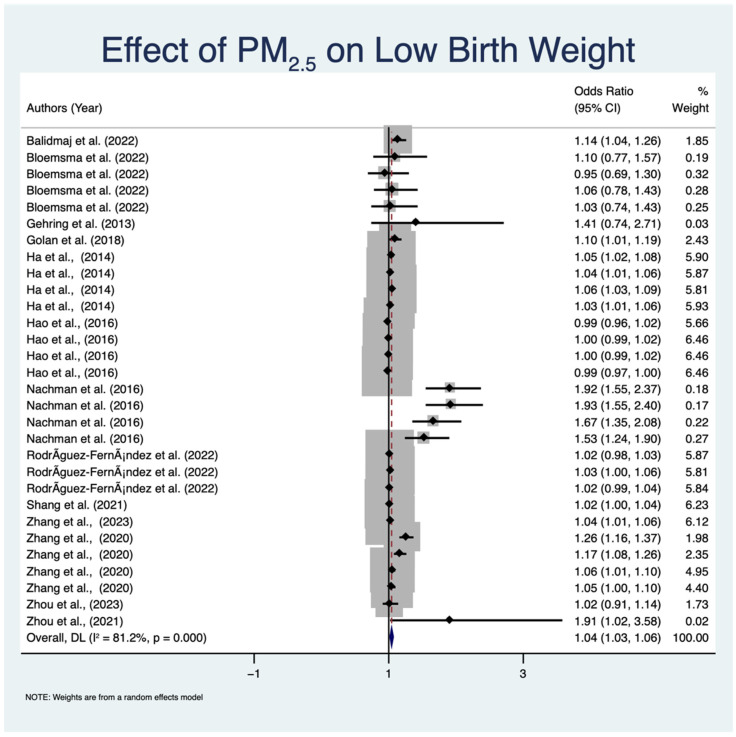
Meta-analysis results of the relationship between LBW and PM_2.5_ exposure. The position of the central point on each gray square indicates the size of the effect for that study [27,28,29,30,31,32,33,34,35,36,37,38,39]. Not the sample size but the weight of the study in this meta-analysis is indicated by the size of each square. The 95% CIs for the effect sizes of each study are shown by horizontal lines through the squares. The hollow diamond shapes indicate the summary effect for each pollutant; the pooled OR across studies is indicated by the diamond’s center, and the pooled 95% confidence interval is shown by its width. The percentage of overall variability across trials caused by heterogeneity rather than chance is shown by the I^2^ statistic. An OR of 1.0 is represented by the dashed red line.

**Figure 3:**
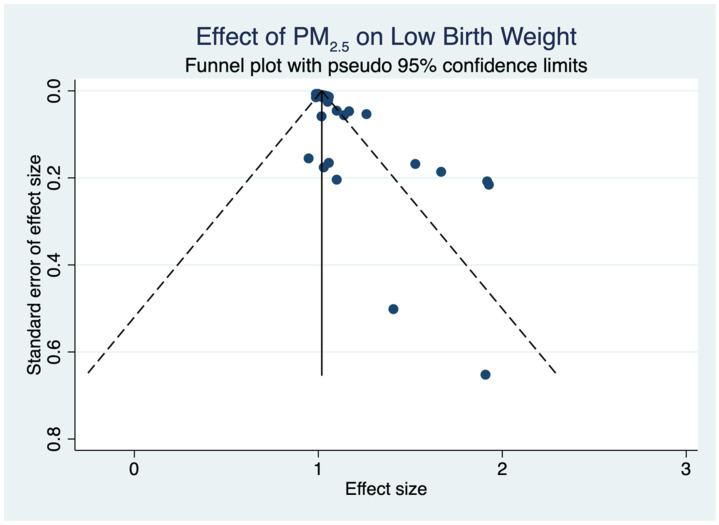
Funnel plot of the relationship between PM_2.5_ and LBW. The solid vertical line represents the overall effect estimate, while the dotted lines indicate the pseudo 95% confidence limits.

Figure 2 demonstrates the results of the effect of PM_2.5_ exposure on LBW across all the included studies in this meta-analysis. Based on the pooled OR of 1.04 (95% CI: 1.03 to 1.06, *p* < 0.001), there is a consistent and moderate correlation between PM_2.5_ exposure and an increased risk of LBW. The substantial heterogeneity observed among the studies (I^2^ = 81.2%, *p* < 0.001) indicates that the impact of PM_2.5_ on LBW varies significantly across the studies included in this meta-analysis. Figure 3 illustrates the publication bias in the studies examining the relationship between PM_2.5_ and LBW. The plot indicates the actual effect size and its standard error, which is represented by 95% CIs. In the absence of heterogeneity or bias, the distribution of studies would exhibit symmetry. The plot depicted in Figure 3 shows a degree of asymmetry, which could indicate publication bias. Despite the indicated heterogeneity, the funnel plot and Egger’s test results show no obvious systematic bias. This provides additional validation for the meta-analytic results presented in Figure 2. The overall findings suggest a relationship between exposure to PM_2.5_ during pregnancy and a higher risk of LBW. Although the pooled OR of 1.04 is small, it has significant public health implications because of its extensive exposure to PM_2.5_ and the critical importance of LBW as a health outcome. Because of the prevalence of PM_2.5_ exposure, even a slight increase in LBW risk can have a significant impact on a large number of children. As a result, the findings emphasize the importance of implementing effective air quality governance strategies and treatments to reduce PM_2.5_ exposure, particularly among expectant mothers.

To evaluate the possibility of publication bias in the selected papers, a funnel plot was plotted and visually examined. An asymmetrical funnel plot suggests the existence of publication bias, wherein smaller studies with non-significant results are less likely to be published. Upon conducting a visual examination, it was seen that the funnel plot displayed several irregularities, indicating the possibility of publication bias. This finding was additionally corroborated by Egger’s regression test, which revealed a statistically significant bias (*p* < 0.05). The presence of an asymmetry in the funnel plot indicates a potential overrepresentation of studies in the literature that report significant relationships between PM_2.5_ exposure and LBW. Several studies included in this meta-analysis, such as Rodríguez-Fernández et al. [27], Zhang et al. [28], and Golan et al. [29], demonstrated a strong correlation between exposure to PM_2.5_ and LBW, potentially influencing the observed bias in the published findings. These studies were conducted using a substantial number of participants and rigorous research methods. However, it is possible that their noteworthy findings may have resulted in an excessive representation of favorable outcomes in the analysis. The studies by Zhang et al. [33] and Zhou et al. [32], which received high scores of nine on the NOS, found a significant relationship and could potentially lead to publication bias. Measurement bias could have an impact on the results. Variability in the results can arise because of inconsistencies in measuring PM_2.5_ exposure and LBW across different studies. The reliability of research findings can be influenced by several approaches to exposure assessment, such as self-reported data and air quality monitoring, as well as different ways of outcome measurement, such as medical records and birth registries. Furthermore, confounding bias arises when the apparent correlation between PM_2.5_ and LBW is affected by unaccounted variables in the studies. Maternal age, socioeconomic level, and access to healthcare are factors that could potentially influence the results and need to be taken into account as confounding variables.

**Figure 4:**
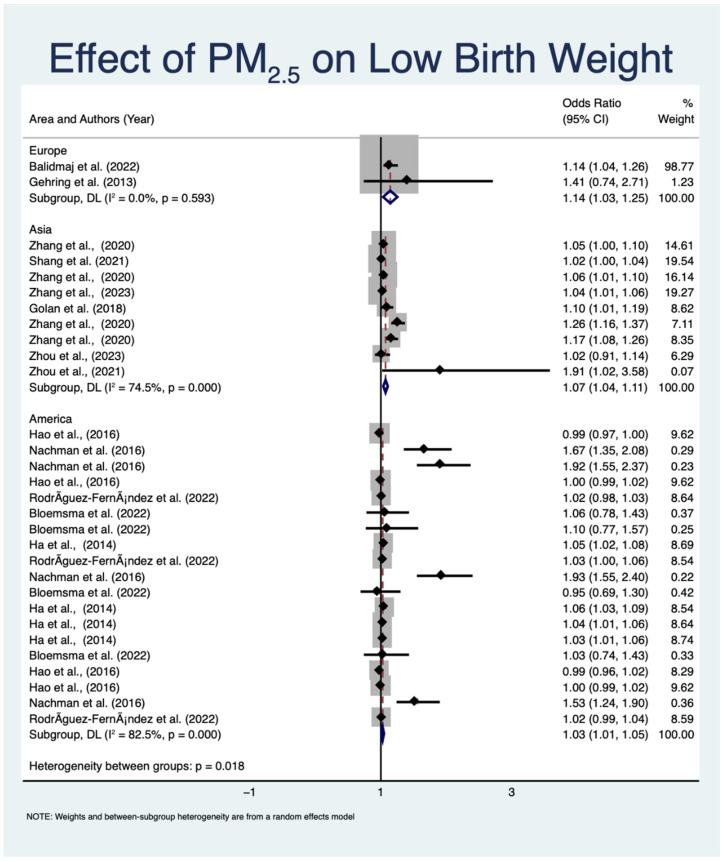
Meta-analysis results of the relationship between LBW and PM_2.5_ exposure across various geographical locations. The position of the central point on each gray square indicates the size of the effect for that study [27,28,29,30,31,32,33,34,35,36,37,38,39]. Not the sample size but the weight of the study in this meta-analysis is indicated by the size of each square. The 95% confidence intervals (CIs) for the effect sizes of each study are shown by horizontal lines through the squares. The hollow diamond shapes indicate the summary effect for each pollutant; the pooled OR across studies is indicated by the diamond’s center, and the pooled 95% confidence interval is shown by its width. The percentage of overall variability across trials caused by heterogeneity rather than chance is shown using the I^2^ statistic. An OR of 1.0 is represented by the dashed red line.

Figure 2 shows significant heterogeneity (I^2^ = 81.2%, *p* < 0.001), indicating variation in the effect estimates. Variations in study designs, demographics, methods of exposure assessment, and geographical locations are likely to affect the variability in study outcomes. For example, differences in pollution levels, socioeconomic circumstances, availability of healthcare, and genetic predispositions in various regions may contribute to the reported discrepancies. Additional subgroup analyses were employed to identify sources of heterogeneity and acquire a more nuanced understanding of the factors influencing the relationship between PM_2.5_ and LBW, as shown in Figure 4. The level of variability shown in Figure 4 is very low in the European studies (I^2^ = 0.0%, *p* = 0.593) but significant in the Asian (I^2^ = 74.5%, *p* < 0.001) and American studies (I^2^ = 82.5%, *p* < 0.001). The studies are categorized by geographical region into three groups as follows: Asia, Europe, and America. The subgroup analysis reveals contradictory odds ratios (ORs) in the relationship between PM_2.5_ exposure and LBW; however, most studies indicate a positive association, with CIs rarely surpassing the null value (OR = 1). In the Europe subgroup, the studies by Balidemaj et al. [37] and Gehring et al. [31] show a positive association with pooled odds ratios of 1.14 (95% CI: 1.03 to 1.25, *p* < 0.001), which means that PM_2.5_ exposure is statistically linked to a much higher risk of LBW. The subgroup exhibits negligible heterogeneity (I^2^ = 0.0%, *p* = 0.593). Several studies in the Asia subgroup, including those by Golan et al. [29], Shang et al. [35], and Zhou et al. [36], have an aggregated OR of 1.07 (95% CI: 1.04 to 1.11, *p* < 0.001), indicating a consistent positive association between PM_2.5_ exposure and LBW. This subgroup demonstrates considerable heterogeneity (I^2^ = 74.5%, *p* < 0.001), suggesting that the study outcomes are subject to variation. A pooled OR of 1.03 (95% CI: 1.01 to 1.05, *p* < 0.001), which comes from studies performed in the America subgroup by Bloemsma et al. [39], Hao et al. [38], and Nachman et al. [30], also shows a positive correlation. Significant heterogeneity exists within this subgroup (I^2^ = 82.5%, *p* < 0.001), indicating that the studies’ findings may vary. Based on the analysis, the pooled ORs for the association between PM_2.5_ and LBW are 1.04 (95% CI: 1.03 to 1.06, *p* < 0.001). Notably, there is substantial heterogeneity (I^2^ = 81.2%, *p* < 0.001), indicating that while there is a consistent association across various regions, there is variability among the individual studies. With a *p*-value of 0.018, the heterogeneity between groups is statistically significant, suggesting that the impact of PM_2.5_ on LBW might differ based on geographic location.

Within the Europe subgroup, the *p*-value of 0.593 suggests that there is no statistically significant correlation between exposure to PM_2.5_ and LBW. This finding may be attributed to several factors. Firstly, Europe generally has stricter air quality regulations and lower average PM_2.5_ concentrations compared with other regions such as Asia and North America. The lower levels of PM_2.5_ exposure may reduce the detectable impact on LBW. Furthermore, the smaller sample sizes or fewer studies from Europe in our meta-analysis could have resulted in a lower statistical power to detect a significant association. These factors, combined with potential differences in genetic susceptibility and lifestyle factors, may contribute to the observed lack of statistically significant correlation in the Europe subgroup. The CI for the Europe subgroup is (1.034, 1.253), indicating that the OR is greater than 1 and suggesting a positive relationship. However, the lack of statistical significance suggests that this result may be due to random variation. A value of 0% for the I^2^ statistic indicates no significant heterogeneity within the Europe subgroup. This suggests that the studies in this subgroup are consistent in their conclusions. Although the OR suggests a possible positive impact, the *p*-value indicates that this impact is not statistically significant. This could be due to a limited number of studies within the Europe subgroup or insufficient statistical power. The Europe subgroup is homogeneous (I^2^ = 0%), indicating high consistency across studies. Nevertheless, this does not imply that the magnitude of the effect is statistically significant. Although the Europe subgroup did not have a substantial impact, the Asia and America subgroups exhibited significant correlations, as indicated by *p*-values below 0.0001, providing strong evidence of a relationship in those regions. These results suggest that variations in PM_2.5_ exposure levels, demographic features, and environmental factors primarily cause heterogeneity in the association between prenatal PM_2.5_ exposure and LBW found in different locations. Previous studies [28,29] support this finding by indicating that differences in air pollution levels, socioeconomic conditions, healthcare availability, and genetic predispositions among different regions can impact the effects of PM_2.5_ on birth consequences.

Variability in results is frequently attributed to the inclusion of studies from different geographical locations, which increases heterogeneity in meta-analyses. The observed variability could potentially be attributed to regional variations in environmental conditions, population demography, and genetic factors [28,29]. Environmental disparities have a substantial impact on fluctuations in PM_2.5_ exposure and its consequences. North America and Europe often enforce more stringent air quality rules than many regions in Asia, leading to lower average levels of PM_2.5_. Nevertheless, urban areas in every location have the potential to experience increased pollution levels because of traffic emissions, industrial activity, and a variety of other sources. Chongqing, China, is an industrial city that has made major efforts to improve air quality. It provides a unique study environment that is distinct from those found in Europe and North America. This demonstrates how pollution levels can vary greatly in different regions [36]. Observed variability is also influenced by demographic disparities, such as inequalities in socioeconomic position, healthcare accessibility, and population density. Populations in certain Asian countries may have elevated levels of PM_2.5_ exposure because of densely populated urban areas with significant industrial emissions, vehicular traffic, and other urban activities. In contrast, rural areas in Europe may experience lower PM_2.5_ levels, with air pollution primarily stemming from agricultural activities, such as the use of fertilizers and the burning of agricultural waste. These demographic and environmental differences can restrict access to healthcare and resources for reducing pollution in certain regions. Conversely, populations in North America and Europe frequently benefit from better healthcare infrastructure and higher socioeconomic status, which may help alleviate certain negative consequences of PM_2.5_ exposure [20]. Policy disparities exacerbate regional variations. North America and Europe have enforced rigorous air quality regulations and measures to decrease emissions, including the implementation of the Clean Air Act in the United States and the air quality directives of the European Union. These efforts have resulted in substantial decreases in PM_2.5_ concentrations in recent decades. On the other hand, several Asian countries are now in the process of creating and implementing thorough air quality control policies. As a result, certain places in these countries experience elevated levels of PM_2.5_ exposure [20,36].

Furthermore, differences in the composition and concentration of air pollution across regions can have a significant impact on the results of studies. It is well-established that air pollution levels and composition vary geographically. For example, PM_2.5_ concentrations in urban regions of Asia, where air pollution is caused by industrial contaminants, may be comparatively higher in weight than in rural areas of Europe, where air pollution is primarily caused by agricultural activities. Regional variations may influence the extent to which PM_2.5_ contributes to adverse health effects, including LBW. Volk et al. [42] argue that differences in air pollution exposure across regions have a substantial impact on the onset of health conditions, including those that influence birth outcomes. Environmental factors such as climate, altitude, and local air quality management policies may all contribute to regional disparities. For example, areas governed by rigorous air quality regulations might encounter reduced concentrations of pollutants, potentially mitigating detectable health consequences. The significance of considering regional variations in air quality and their potential ramifications on health outcomes, such as respiratory and developmental disorders, is emphasized by Šrám et al. [43]. Further heterogeneity may be introduced by variations in study designs across regions, in addition to environmental factors. Because of their longitudinal design, prospective cohort studies, which follow individuals from the moment of exposure to the outcome, are generally regarded as more methodologically rigorous. This method permits the gradual accumulation of more precise and exhaustive data. Raz et al. [20] state that the Nurses’ Health Study II is a commendable prospective cohort study that adequately tracks the long-term changes in environmental exposures and their effects on health. On the other hand, retrospective studies are susceptible to biases and inaccuracies because of their reliance on pre-existing records and participant recollections. This approach introduced the possibility of recall bias and data inconsistencies. The heterogeneity observed in our meta-analysis results can be attributed to the inclusion of studies from diverse geographical regions and varying methodological approaches. This presents difficulties in formulating consistent conclusions. The variations in methodology and geography highlight the criticality of regional contexts and study designs in the interpretation of our meta-analytical results.

In summary, the results of this meta-analysis indicate a consistent relationship between perinatal PM_2.5_ exposure and an increased risk of LBW, as illustrated in Figure 4. The analysis indicates a strong correlation between PM_2.5_ exposure during pregnancy and increased ORs for LBW. The heterogeneity among subgroups specific to geographical regions suggests that several factors influence the occurrence of LBW. Variability in these effects can be attributed to differences in the examined populations, environmental conditions, and exposure estimation methodologies. In addition, the duration of exposure is crucial for determining the risk of LBW. Higher ORs were reported in studies conducted in various regions, including those by Zhou et al. [38] and Golan et al. [31], confirming the association between prenatal PM_2.5_ exposure and LBW. The results exhibit heterogeneity, particularly among the subgroups representing Asia and America. This suggests that regional and environmental conditions exert a substantial influence on LBW. The range of relationships observed with LBW may have also been influenced by the utilization of different assessment tools across studies. An example of this is the potential for accuracy variation when estimating PM_2.5_ exposure using land-use regression models, air quality monitoring data, or satellite-based exposure methodologies to estimate PM_2.5_ exposure. Variables in exposure assessment methods and reported prevalence may influence associations and correlations with LBW.

#### 3.4.2. Effect of PM_2.5_ Exposure on LBW across Different Trimesters

**Figure 5:**
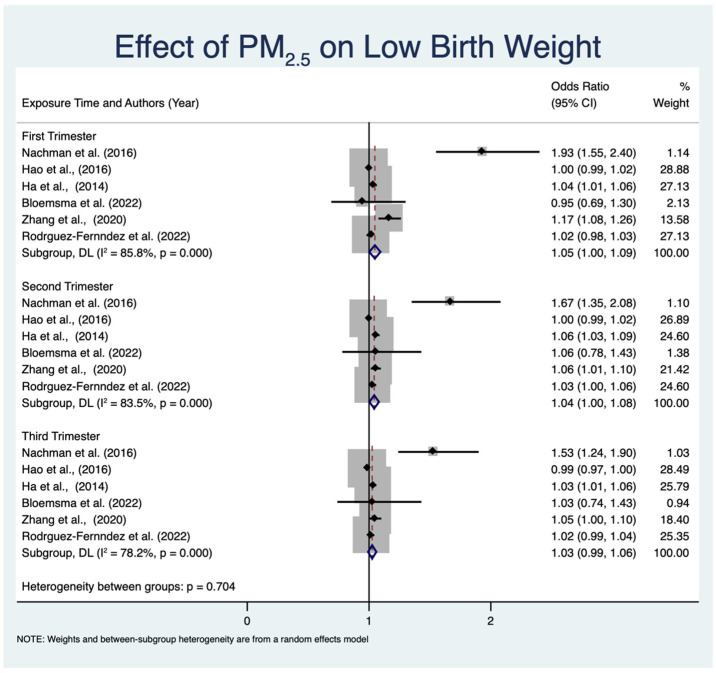
The association between PM_2.5_ exposure and LBW across different trimesters [27,28,30,34,38,39].

As shown in previous analyses, exposure to PM_2.5_ during pregnancy has been associated with negative effects on LBW. The effect of this exposure, however, differs depending on the specific phase of pregnancy [44]. This section analyzes the impact of PM_2.5_ exposure on LBW throughout the first, second, and third trimesters. It aims to provide a thorough understanding of how the timing of exposure affects the growth of the fetus. The forest plot in Figure 5 displays the correlation between PM_2.5_ exposure and LBW during various trimesters. This study categorizes the data into three subgroups as follows: the first trimester, the second trimester, and the third trimester. This meta-analysis examined the influence of PM_2.5_ exposure on the occurrence of LBW at different stages of pregnancy. This study shows that there were significant relationships between exposure to PM_2.5_ and LBW during the first (OR 1.05, 95% CI: 1.00–1.09, *p* < 0.001) and second trimesters (OR 1.04, 95% CI: 1.00–1.08, *p* < 0.001). There is no significant variation in the relationships across trimesters (*p* = 0.704). The subgroup analysis of the first trimester demonstrates a pooled OR of 1.05 (95% CI: 1.00 to 1.09, *p* < 0.001), indicating a statistically significant correlation between exposure to PM_2.5_ and LBW. The studies in this category show significant heterogeneity (I^2^ = 85.8%, *p* < 0.001), indicating that the impact of PM_2.5_ on LBW is impacted by different factors in each study. During the second trimester, the combined OR is 1.04 (95% CI: 1.00 to 1.08), indicating a substantial correlation between exposure to PM_2.5_ and LBW. The level of heterogeneity in this subgroup is moderately reduced (I^2^ = 83.5%, *p* < 0.001), but it remains significant. This indicates that although the second trimester is a critical stage for the development of the fetus, the differences in how studies are conducted and the characteristics of the population being studied still have an impact. The subgroup analysis of the third trimester reveals a pooled OR of 1.03 (95% CI: 0.99 to 1.06, *p* < 0.001), indicating that the results are also statistically significant but smaller than the first and second trimesters. The level of heterogeneity remains significantly high (I^2^ = 78.2%, *p* < 0.001). The lack of a significant correlation in the third trimester may be attributed to the diminished susceptibility of fetal growth to external contaminants in comparison with earlier stages. Nevertheless, prolonged exposure to PM_2.5_ might result in negative consequences for LBW. These effects occur because of processes such as endocrine disruption and epigenetic changes [45].

**Figure 6:**
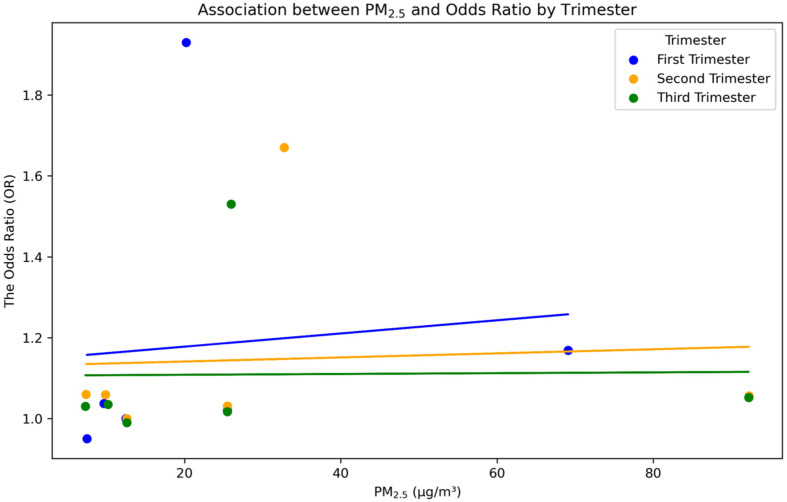
The regression of the association between PM_2.5_ concentrations and the OR for LBW across the first (blue), second (orange), and third (green) trimesters.

For more details on the analysis of the effect of PM_2.5_ exposure on the LBW in several trimesters, Figure 6 depicts a linear regression model establishing the relationship for each trimester. The LBW in each trimester is presented in a scatter plot alongside the associated regression lines. During the first trimester, the scatter plot exhibits a positive correlation between PM_2.5_ concentrations and the OR. These findings indicate that increased PM_2.5_ levels in the first trimester are related to a higher risk of LBW. During the second trimester, the regression line likewise shows a positive correlation, albeit with a less steep slope compared with the first trimester. This suggests that although there is a correlation between exposure to PM_2.5_ and LBW, the impact is less pronounced than in the first trimester. During the third trimester, the regression line tends to have a relatively low slope, suggesting a weak correlation between PM_2.5_ concentrations and the OR. These findings indicate that the influence of PM_2.5_ exposure on LBW is less pronounced during the third trimester compared with the previous trimesters.

The results of the analyses in Figure 5 and Figure 6 suggest that PM_2.5_ exposure during the first three months of pregnancy is related to an increased risk of LBW. The increased vulnerability could be attributed to a variety of critical factors influencing embryonic growth in the early stage of gestation [46]. The first three months of pregnancy are a critical stage for the growth of the fetus, as they are characterized by rapid cell multiplication, the formation of organs, and the creation of the placenta [46]. At this stage, the embryo is highly susceptible to PM_2.5_. Disruptions occurring in the developmental processes during the first three months of pregnancy might result in enduring consequences for the growth and development of the fetus [47,48]. Exposure to PM_2.5_ during the first three months of pregnancy causes oxidative stress and inflammation, which can harm the health of both the mother and the developing fetus [49]. Inhalation of PM_2.5_ particles might result in the generation of reactive oxygen species (ROS), which cause oxidative damage in maternal tissues [50]. Oxidative stress causes inflammatory reactions, which are marked by the secretion of pro-inflammatory cytokines like interleukin-6 (IL-6) and tumor necrosis factor-alpha (TNF-α) [51,52]. The inflammatory mediators could pass through the placental barrier, affecting the growth of the developing baby and perhaps limiting its growth [51,52]. The placenta is essential for facilitating the transfer of nutrients and oxygen between the mother and fetus [51,52]. Exposure to PM_2.5_ has been linked to placental disruption, which can potentially undermine this crucial function [50]. PM_2.5_ could change the structure and functioning of the placenta, resulting in decreased blood flow and nutritional transportation in the placenta [53]. PM_2.5_ causes inflammation and oxidative stress in the placenta, which can lead to damage in the placental blood vessels [54]. This damage restricts the delivery of important nutrients and oxygen to the fetus, resulting in LBW [55,56]. PM_2.5_ particles can also function as endocrine disruptors, impeding hormonal pathways that are crucial for the maintenance of pregnancy and the development of the fetus [57]. These particles could modify the amounts of hormones, such as cortisol and insulin, that are essential for the growth of the fetus. Disturbance of the maternal endocrine system affects fetal growth and development, ultimately leading to LBW [17,58]. Recent studies indicate that exposure to PM_2.5_ may cause epigenetic alterations, which are heritable adjustments in gene expression that do not modify DNA sequences. Epigenetic changes, such as DNA methylation and histone modification, can impact fetal growth patterns and developmental processes. Alterations in the epigenetic modifications of important genes that control fetal growth can lead to negative birth outcomes, such as LBW [47,59]. In addition, exposure to PM_2.5_ might result in maternal and fetal hypoxia, which is a state characterized by inadequate oxygen delivery [60]. Inhaling tiny particulate matter might hinder the respiratory function of pregnant women, leading to decreased oxygen levels in the bloodstream [61]. The lack of oxygen in this condition might harm the fetus, as sufficient oxygen is crucial for proper growth and development. Prolonged oxygen deprivation in the fetus can result in LBW [61,62].

The effects of exposure to PM_2.5_ differ depending on the trimester of pregnancy because of distinct biological mechanisms [44]. In the first trimester, crucial biological processes include the implantation of the fertilized egg into the uterine lining, the formation of the placenta, and the development of organs [63]. Exposure to PM_2.5_ during this period can interfere with these processes by causing oxidative stress and inflammation, which can impact cellular structures and normal placental function [55,64]. During the second trimester, the fetus experiences accelerated growth and ongoing placental development, which heightens its susceptibility to environmental contaminants [65]. Exposure to PM_2.5_ at this time can result in placental insufficiency, which decreases the availability of oxygen and nutrients to the fetus. This can lead to restricted growth and LBW [66,67]. During the third trimester, the main emphasis is on the growth and maturity of fetal organs [68]. However, it is important to note that the fetus is still susceptible to the detrimental impact of PM_2.5_. Nevertheless, this meta-analysis did not discover a substantial correlation between exposure to PM_2.5_ during the third trimester and LBW. This lack of a link may be attributed to the fetus’s heightened ability to withstand the effects of PM_2.5_ or its compensatory development mechanisms that are active during this stage [69,70]. Compensatory growth mechanisms entail an improvement in nutrient absorption and utilization, which aids in the development of the fetus even in unfavorable circumstances.

#### 3.4.3. Biological Pathways of PM_2.5_ Exposure in Pregnancy and LBW

The results of this meta-analysis identify significant associations between prenatal PM_2.5_ exposure and LBW, particularly in the first and second trimesters. The observed associations are consistent with the existing literature that proposes potential biological mechanisms, such as oxidative stress, inflammation, placental dysfunction, endocrine disruption, and epigenetic modifications. However, it is important to note that this meta-analysis does not establish causality or directly test these mechanisms. The existing literature suggests that oxidative stress, inflammation, placental dysfunction, endocrine disruption, and epigenetic changes might play crucial roles, but these hypotheses need direct testing through experimental or longitudinal studies. Prenatal exposure to PM_2.5_ can result in reduced birth weight through various inter-connected mechanisms. The pathway of oxidative stress and inflammation is initiated by exposure to PM_2.5_, leading to the production of ROS [71]. These ROS induce oxidative stress and activate inflammatory reactions. Inflammatory mediators, such as tumor necrosis factor-alpha (TNF-α), and interleukin-6 (IL-6) could pass through the placental barrier and negatively affect the development of the fetus, resulting in LBW [72]. The pathway of placental dysfunction is characterized by inflammation and oxidative stress generated by PM_2.5_, which impair the placental vasculature and reduce the effectiveness of nutrient and oxygen transportation to the fetus [73]. This malfunction hampers fetal growth and adds to the condition of LBW. PM_2.5_ particles function as endocrine disruptors in the endocrine disruption pathway, modifying hormone levels that are essential for sustaining pregnancy and facilitating fetal growth [74]. Interference with various hormonal pathways, such as cortisol, estrogen, progesterone, and insulin, might hinder fetal development, leading to a lower-than-average birth weight [75]. The pathway of epigenetic modifications emphasizes the ability of PM_2.5_ exposure to cause changes in gene expression patterns that are vital for embryonic growth through processes such as DNA methylation and histone modification [75]. Epigenetic changes have the potential to result in limited fetal growth and reduced birth weight [75]. The hypoxia pathway demonstrates how exposure to PM_2.5_ might hinder the respiratory function of mothers, resulting in decreased oxygen levels in the bloodstream [73]. The hypoxic situation in the fetus leads to LBW because of inadequate oxygen supply [73]. Together, these pathways emphasize the several complex processes by which exposure to PM_2.5_ negatively impacts the development of a fetus and the significance of reducing air pollution to support positive pregnancy outcomes [71].

The pathway of oxidative stress and inflammation is a key mechanism by which PM_2.5_ affects the development of the fetus [51,52]. When PM_2.5_ particles are breathed in, they enter the respiratory system and travel deep into the alveoli, where they might move into the bloodstream [76]. This leads to the generation of ROS, which are highly reactive molecules capable of causing cellular and molecular damage. Oxidative stress occurs when the body’s antioxidant defenses are unable to cope with the excessive creation of ROS [51,52]. The initiation of nuclear factor-kappa B (NF-κB) and the subsequent production of pro-inflammatory cytokines such as interleukin-6 (IL-6) and tumor necrosis factor alpha (TNF-α) are the characteristic features of the inflammatory responses induced by aerobic stress [51,52]. Cytokines can traverse the placental barrier and enter the fetal circulation, resulting in the development of an inflammatory milieu that can hinder fetal growth. Inflammation can also cause placental dysfunction by altering blood vessels in the placenta and decreasing the transfer of nutrients and oxygen to the fetus. The concurrent occurrence of oxidative damage and inflammation generates an antagonistic intrauterine milieu that hampers fetal development, ultimately resulting in reduced birth weight. Research has demonstrated that pregnant women who are exposed to high levels of PM_2.5_ and have increased levels of inflammatory markers are more likely to give birth to babies with lower birth weights [51,56,71]. This pathway emphasizes the need to tackle air pollution to prevent negative pregnancy outcomes and the necessity for additional study to understand the biological mechanisms involved completely.

The pathway of placental dysfunction is a critical mechanism by which prenatal exposure to PM_2.5_ might result in reduced birth weight [53]. The placenta is essential during pregnancy as it enables the transfer of nutrition, oxygen, and waste products between the mother and fetus [53]. PM_2.5_ particles, when breathed in, can enter the maternal bloodstream and then reach the placenta [51,52,53,76]. This exposure can trigger inflammatory reactions and oxidative stress in the placental tissue. Oxidative stress arises from an inequilibrium between the generation of ROS and the placenta’s ability to remove these reactive chemicals, leading to cellular damage. Placental inflammation is marked by heightened production of pro-inflammatory cytokines, including interleu-kin-6 (IL-6) and tumor necrosis factor-alpha (TNF-α) [51,52]. The presence of these inflammatory mediators can negatively affect the physical and functional health of the placenta, causing disruptions in its vascular system [53]. This interruption decreases the flow of blood to the placenta, which in turn restricts the delivery of vital nutrients and oxygen to the growing fetus. Moreover, exposure to PM_2.5_ might modify the structure of the placenta, leading to a decrease in the thickness of the trophoblast layer and a reduction in the creation of villi, which are essential for the absorption of nutrients [53]. The placenta’s capacity to adequately sustain fetal growth is compromised by the cumulative impact of oxidative stress, inflammation, and structural alterations. Consequently, the fetus undergoes IUGR, a syndrome strongly associated with LBW. A study showed that the exposure of pregnant women to high levels of PM_2.5_ can cause major changes in the way the placenta functions as well as its structure. This can result in negative effects on the birth outcomes of babies [55,56,71]. This pathway emphasizes the significance of reducing air pollution exposure during pregnancy to ensure normal placental function and enhance healthy fetal development.

Prenatal exposure to PM_2.5_ can lead to LBW through the endocrine disruption route, which is a significant mechanism. PM_2.5_ particles can transport various harmful compounds, such as polycyclic aromatic hydrocarbons (PAHs), heavy metals, and persistent organic pollutants (POPs), which can function as disruptors of the endocrine system [45,55]. These drugs have the potential to disrupt the normal hormonal signaling pathways that are crucial for maintaining pregnancy and supporting the growth of the fetus [71]. When these endocrine-disrupting chemicals (EDCs) enter the bloodstream of the mother, they can change the levels and functioning of important hormones such as cortisol, estrogen, progesterone, and insulin. For instance, increased concentrations of cortisol, a hormone associated with stress, can decrease the flow of blood to the placenta and hinder the transfer of nutrients, therefore affecting the growth of the fetus [77]. Disturbances in estrogen and progesterone signaling can impact the formation of the placenta and its capacity to sustain the fetus [78]. Furthermore, modified insulin signaling can influence the metabolic processes and development of the fetus. Disruption of these hormonal pathways can result in poor placental function, insufficient provision of nutrients and oxygen to the fetus, and, ultimately, IUGR, which is characterized by LBW. A study indicated that the exposure of mothers to endocrine-disrupting chemicals (EDCs) might lead to negative effects on pregnancy, such as lower birth weights and higher chances of giving birth prematurely [45,55,71]. These findings emphasize the importance of endocrine disruption as a method by which exposure to PM_2.5_ negatively impacts fetal development. This highlights the necessity of implementing strategies to decrease exposure to dangerous air pollutants while pregnant.

The cascade of epigenetic changes is a vital mechanism by which prenatal exposure to PM_2.5_ might result in reduced birth weight. Epigenetic changes refer to alterations in gene expression that can be passed down from one generation to another and happen without any mutations in the DNA sequence. These changes involve DNA methylation, modification of histones, and expression of non-coding RNA. PM_2.5_ particles can transport various harmful compounds that can disrupt the typical epigenetic control of genes that are essential for the development of a fetus [45]. Inhaling these particles can cause systemic oxidative stress and inflammation, leading to changes in the epigenetic profile of both maternal and fetal tissues. For example, elevated amounts of ROS and pro-inflammatory cytokines can impact the patterns of DNA methylation, resulting in the suppression of genes essential for the proper growth of the placenta and fetus [64]. One study demonstrated that exposure to elevated levels of PM_2.5_ is linked to a decrease in global DNA methylation and an increase in methylation of genes related to growth regulation and metabolic pathways [45]. Epigenetic modifications have the potential to disturb regular cellular processes and hinder the development of the fetus by modifying the activity of genes that control the transportation of nutrients, cellular growth, and specialization. Moreover, these epigenetic alterations could endure beyond the period of exposure, thereby exerting lasting impacts on the health and development of the fetus [41,53]. The study conducted by Janssen et al. [45] revealed that exposure to particle air pollution is directly linked to placental DNA hypomethylation. This suggests that environmental pollutants have an impact on fetal growth through epigenetic modifications. In addition, research conducted by van den Hooven et al. [55] and Gluckman and Hanson [48] emphasized the crucial function of epigenetic pathways in influencing the impact of environmental exposures on birth outcomes. These findings emphasize the significance of comprehending epigenetic alterations as a mechanism by which PM_2.5_ exposure negatively impacts fetal development.

The hypoxia pathway is a major route by which prenatal exposure to PM_2.5_ might result in reduced birth weight. Hypoxia is a state characterized by an inadequate provision of oxygen to bodily tissues. Inhaling PM_2.5_ particles can lead to respiratory and cardiovascular dysfunctions in pregnant women, which can decrease the effectiveness of oxygen transport from the mother’s blood to the placenta and fetus [61,62]. A decreased supply of oxygen can directly affect the growth and development of the fetus. PM_2.5_ particles, which frequently contain harmful chemicals including heavy metals and organic compounds, can cause widespread inflammation and oxidative stress in pregnant women [45,56]. These disorders can hinder the functioning of the lungs, resulting in a decrease in the amount of oxygen that is taken in [51]. Furthermore, inflammation and oxidative stress might impact the cardiovascular system, leading to reduced blood circulation and diminished oxygen supply to the placenta. PM_2.5_ exposure can directly impact the placenta, which is responsible for facilitating the flow of nutrients and oxygen between the mother and fetus. Research has demonstrated that PM_2.5_ can induce inflammation in the placenta and impair its ability to provide sufficient oxygen to the fetus, leading to vascular dysfunction [45,56]. Chronic prenatal hypoxia elicits many adaptive reactions in the fetus to preserve oxygen for essential organs like the brain and heart. These responses result in decreased rates of growth and changes in metabolic processes, which can cause LBW. Studies have shown that hypoxia can hinder the activation of genes that promote growth and trigger pathways that respond to stress, resulting in impaired fetal growth [47,54]. In addition, hypoxia can impact the epigenetic control of genes that are crucial for the development of the fetus. Studies have demonstrated that hypoxic circumstances can modify DNA methylation and histone modification patterns, resulting in persistent alterations in gene expression that may contribute to negative birth outcomes [45]. These alterations in gene expression can sustain limitations in growth and enhance vulnerability to long-term chronic illnesses.

### 3.5. Limitation of this Study

This study effectively combines rigorous methodological techniques, a thorough literature assessment, strong statistical analysis, and an in-depth examination of biological systems. These combined qualities significantly improved the reliability, dependability, and significance of the results indicating the influence of prenatal PM_2.5_ exposure on low birth weight. However, it is important to consider the limitations of this study while analyzing the results. The included studies exhibit substantial variety in terms of research design, population characteristics, and methodologies used to quantify PM_2.5_ exposure. This heterogeneity has the potential to influence the comparability of outcomes and generate bias. Subsequent investigations should focus on establishing uniform methodologies for evaluating exposure and include stratified analyses to accommodate diverse demographic groupings. Furthermore, it is important to acknowledge that there is a possibility of residual uncertainty that cannot be eliminated. Although we accounted for significant variables such as maternal age, socioeconomic position, and smoking, it is important to note that additional factors such as nutrition, pre-existing health issues, and access to prenatal care could possibly impact the observed relationships. To strengthen the reliability of the results, future research should incorporate more extensive data on these supplementary factors that may influence the outcomes. In addition, the utilization of observational studies in this meta-analysis implies that the establishment of causation is not conclusively possible. Conducting randomized controlled trials (RCTs) to study environmental exposures is not practical. However, future research could utilize quasi-experimental techniques, such as natural experiments or instrumental variable analyses, to enhance the accuracy of causal conclusions. Moreover, the issue of publication bias arose, as research studies that have noteworthy results are more inclined to be published. This was demonstrated by the lack of symmetry in the funnel plot and the findings of Egger’s regression test. To tackle this problem in the future, researchers could adopt the practice of pre-registering study protocols and promoting the publication of all findings, irrespective of their significance. In addition, numerous studies included self-reported data for birth outcomes, which may lead to recall bias and misclassification. To obtain a more precise and dependable assessment of outcomes, future research should employ objective measures, such as medical records or birth registries. These constraints can have an impact on the findings of this study and how they are interpreted. The presence of high heterogeneity and the possibility of residual confounding indicate that the observed relationships may not be solely due to exposure to PM_2.5_. Publication bias suggests that there is a possibility of overestimating the actual effect size. These considerations emphasize the need to interpret our results carefully and the importance of conducting more studies to confirm our findings.

## 4. Conclusions

This study presents the findings of a systematic review and meta-analysis that investigate the relationship between prenatal exposure to PM_2.5_ and LBW. This study examines multiple trimesters involved in the relationship. The results indicate a consistent relationship between prenatal exposure to PM_2.5_ and an increased risk of LBW, with the effects being most pronounced in the first and second trimesters.

## Figures and Tables

**Table 1:** Selected studies on the effect of PM_2.5_ on LBW.

No.	Author (Year)	Country (Study Design)	Number of Participants	Assessment Tool	Estimated PM_2.5_ Concentration	Air Pollution Exposure Period	Exposure Assessment	Main Finding
1	Rodríguez-Fernández et al. [27]	Chile (cross-sectional study)	595,369	Low birth weight	25.5 µg/m^3^ to 55.7 µg/m^3^	Specific trimesters	Air Quality Information System data	Maternal intake of PM_2.5_ within the second trimester of pregnancy increases the probability of giving birth to a baby with LBW.
2	Zhang et al. [28]	China (cohort study)	3369	Low birth weight	58.53 µg/m^3^ to 129.53 µg/m^3^	Entire pregnancy	Land-use regression models	There is a strong correlation between PM_2.5_ and LBW.
3	Golan et al. [29]	Israel (cohort study)	750,000	Low birth weight	12.5 µg/m^3^ to 25.1 µg/m^3^	Entire pregnancy	Air pollution monitoring data	There is a direct correlation between exposure to PM_2.5_ and LBW.
4	Nachman et al. [30]	USA (cohort study)	5059	Low birth weight	5.54 µg/m^3^ to 29 µg/m^3^	Entire pregnancy	National Environmental Public Health Tracking Network data	Exposure to PM_2.5_ is linked to a higher likelihood of LBW.
5	Gehring et al. [31]	Europe (cohort study)	5921	Low birth weight	15.3 µg/m^3^ to 21.1 µg/m^3^	Entire pregnancy	Land-use regression models	Exposure to PM_2.5_ is associated with reduced birth weights.
6	Zhou et al. [32]	China (cohort study)	13,335	Low birth weight	48.6 µg/m^3^ to 85.5 µg/m^3^	Entire pregnancy	Air pollution monitoring data	Elevated susceptibility to reduced birth weight is associated with exposure to PM_2.5_.
7	Zhang et al. [33]	Australia (cohort study)	330,884	Low birth weight	0.5 µg/m^3^ to 4.02 µg/m^3^	Entire pregnancy	Satellite-based exposure methods	A strong correlation exists between PM_2.5_ and LBW.
8	Ha et al. [34]	USA (cohort study)	423,719	Low birth weight	9.7 µg/m^3^ to 10.2 µg/m^3^	Entire pregnancy	National air pollution monitoring network	Exposure to PM_2.5_ is linked to reduced birth weight.
9	Shang et al. [35]	China (cohort study)	536,993	Low birth weight	13.20 µg/m^3^ to 115.36 µg/m^3^	Entire pregnancy	Generalized Additive Model	Exposure to PM_2.5_ is linked to both LBW and preterm birth.
10	Zhou et al. [36]	China (cohort study)	572,106	Low birth weight	17.82 µg/m^3^ to 83.65 µg/m^3^	Entire pregnancy and specific trimesters	Generalized Additive Model	There is a strong correlation between exposure to PM_2.5_ and the occurrence of very LBW.
11	Balidemaj et al. [37]	Sweden (cohort study)	43,256	Low birth weight	0.3 µg/m^3^ to 4.2 µg/m^3^	Entire pregnancy	Land-use regression models	Exposure to PM_2.5_ is associated with reduced birth weight.
12	Hao et al. [38]	USA (cohort study)	3,389,450	Low birth weight	4.7 μg/m^3^ to 23.8 μg/m^3^	Entire pregnancy	Air pollution monitoring data	Exposure to PM_2.5_ is linked to a higher likelihood of LBW.
13	Bloemsma et al. [39]	USA (cohort study)	1410	Low birth weight	6.8 µg/m^3^ to 8.2 µg/m^3^	Entire pregnancy	Air pollution monitoring data	There is a direct correlation between exposure to PM_2.5_ and LBW.

**Table 2:** Quality assessment of the selected study using the Newcastle–Ottawa quality rating scale.

No.	Author (Year)	Selection (Max 4)	Comparability (Max 2)	Outcome (Max 3)	Total Score (Max 9)
1	Rodríguez-Fernández et al. [27]	3	1	2 *	6 *
2	Zhang et al. [28]	4	2	3	9
3	Golan et al. [29]	3	2	3	8
4	Nachman et al. [30]	4	1	2 *	7 *
5	Gehring et al. [31]	3	2	2 *	7 *
6	Zhou et al. [32]	4	2	3	9
7	Zhang et al. [33]	4	2	3	9
8	Ha et al. [34]	4	1	2 *	7 *
9	Shang et al. [35]	4	2	3	9
10	Zhou et al. [36]	4	2	3	9
11	Balidmaj et al. [37]	3	1	2 *	6 *
12	Hao et al. [38]	4	1	2 *	7 *
13	Bloemsma et al. [39]	3	2	2 *	7 *

The score for each area (selection, comparability, outcome) is determined according to certain criteria. Each numbered item within the selection and outcome categories can receive a maximum of one star in a study. The maximum number of stars that may be given for comparability is two. The “*” symbol represents ambiguity in the overall score caused by insufficient data on the duration and sufficiency of follow-up, which impacts the score in the outcome area. A greater overall score indicates a superior evaluation based on the NOS criteria.

## Data Availability

All data generated or analyzed during this study are included in this published article.
